# Hypericins as Potential Leads for New Therapeutics

**DOI:** 10.3390/ijms11020562

**Published:** 2010-02-04

**Authors:** Anastasia Karioti, Anna Rita Bilia

**Affiliations:** Department of Pharmaceutical Sciences, University of Florence, Ugo Schiff 6, Polo Scientifico, Sesto Fiorentino, 50019, Florence, Italy; E-Mail: ar.bilia@unifi.it

**Keywords:** *Hypericum perforatum*, naphthodianthrones, hypericin, pseudohypericin, photodynamic therapy, antiviral, antidepressant

## Abstract

70 years have passed since the first isolation of the naphthodianthrones hypericin and pseudohypericin from *Hypericum perforatum* L. Today, they continue to be one of the most promising group of polyphenols, as they fascinate with their physical, chemical and important biological properties which derive from their unique chemical structure. Hypericins and their derivatives have been extensively studied mainly for their antitumor, antiviral and antidepressant properties. Notably, hypericin is one of the most potent naturally occurring photodynamic agents. It is able to generate the superoxide anion and a high quantum yield of singlet oxygen that are considered to be primarily responsible for its biological effects. The prooxidant photodynamic properties of hypericin have been exploited for the photodynamic therapy of cancer (PDT), as hypericin, in combination with light, very effectively induces apoptosis and/or necrosis of cancer cells. The mechanism by which these activities are expressed continues to be a main topic of discussion, but according to scientific data, different modes of action (generation of ROS & singlet oxygen species, antiangiogenesis, immune responces) and multiple molecular pathways (intrinsic/extrinsic apoptotic pathway, ERK inhibition) possibly interrelating are implicated. The aim of this review is to analyse the most recent advances (from 2005 and thereof) in the chemistry and biological activities (*in vitro* and *in vivo*) of the pure naphthodianthrones, hypericin and pseudohypericin from *H. perforatum*. Extracts from *H. perforatum* were not considered, nor pharmakokinetic or clinical data. Computerised literature searches were performed using the Medline (PubMed), ChemSciFinder and Scirus Library databases. No language restrictions were imposed.

## 1. Introduction

### 1.1. Distribution, Plant Sources

**Figure 1.**
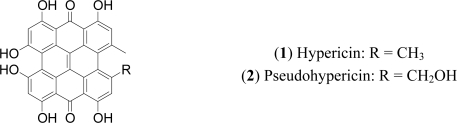
Structures of hypericin and pseudohypericin.

**Figure 2.**
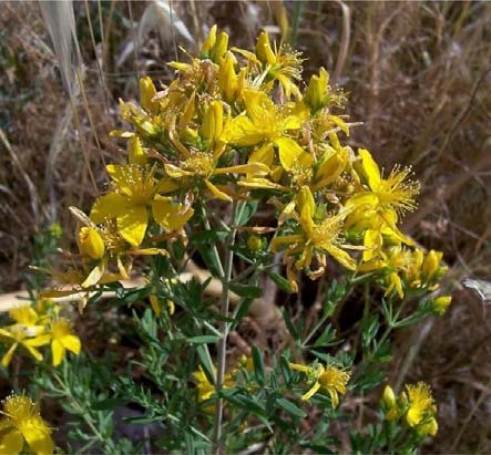
*Hypericum perforatum* L. (From: http://luirig.altervista.org/photos/hypericum_perforatum.htm/. Flora italiana.)

Hypericin (**1**) and pseudohypericin (**2**) are two natural products, structurally belonging to the chemical class of naphthodianthrones (Figure 1) and the characteristic constituents of the genus *Hypericum* (Clusiaceae). Plants of this developed genus (comprising approximately 450 species) are widely distributed all around the world and have been used since antiquity for a range of medicinal properties. Hypericin and pseudohypericin were first isolated from *Hypericum perforatum* L., [1–3], which is the most important and commercially recognized representative of the genus. *H. perforatum*, commonly known as St. John’s Wort, is an aromatic, perennial herb growing up to 1 m high (Figure 2). Its leaves are characterized by the presence of oil glands which may be seen upon holding the leaf to light, giving the impression of a perforated look, to which is attributed the name of the plant. The flowers, are bright yellow-orange, while the calyx and corolla are marked with numerous black glandular dots. When the black dots are rubbed between the fingers, the fingers become red [4,5].

In *H. perforatum*, the naphthodianthrone content ranges from 0.05 to 0.30% [6], but it can vary depending on the cultivar, altitude, light conditions, period of the year [7]. The plant also contains protohypericin, protopseudohypericin and cyclopseudohypericin. All these occur mainly in the flowering parts of the plant and especially located in the dark glands [8]. Protohypericin and protopseudohypericin are considered to be the biosynthetic precursors of hypericin and pseudohypericin, respectively, from which the latter derive on exposure to light [9]. Also pseudohypericin is likely to be transformed to cyclopseudohypericin [10]. Protohypericin, protopseudohypericin and cyclopseudohypericin are detected in lower concentrations and are included in the analytical term “total hypericins” or “total naphthodianthrones” [11]. In general, the content of pseudohyhypericin (0.03–0.34%) is higher from that of hypericin (0.03–0.09%) two to four times [12], depending on the variety.

### 1.2. Other Sources of Hypericin and Pseudohypericin

Hypericin and pseudohypericin are found in several *Hypericum* sp. A recent review by Bruni and Sacchetti [7] shed ample light on the morphological and chemical variations among different *Hypericum* sp. and varieties. Hitherto, only a small part of the genus has been studied, whereas approximately ¾ of *Hypericum* species have not been surveyed for their chemical content. A recent survey on 74 *Hypericum* taxa (approx. 20% of the entire genus) [13] from different continents showed that hypericins were detected only in species of the Sections *Hypericum*, *Adenotras*, *Drosocarpium.* Generally, it seems that their presence is restricted to phylogenetically advanced sections of the genus. Hypericin and pseudohypericin are absent or negligible in the primitive sections, whereas in some taxa (*H. hirsutum*, *H. empetrifolium*) only hypericin can be found [14]. Infrageneric chemotaxonomic surveys, have revealed that some *Hypericum* species, such as *H. boissieri*, *H. barbatum*, *H. rumeliacum* may contain a 2–4 fold higher amount of hypericins than *H. perforatum*. The latter, however, still remains as the sole sourse of *Hyperici Herba*. There many reasons for this: its wide geographical diffusion from temperate to tropical mountain regions, its adaptation to diverse environmental conditions, its cultivation and of course its diffused therapeutic use.

### 1.3. Endophytic Fungi

Besides *Hypericum* sp., hypericin has also been found in fungi such as *Dermocybe* [15] or endophytic fungi which grow in diverse *Hypericum* sp. or in other plant species. The latter source represents a very interesting field in the discovery of alternative ways for the production of hypericin. It has been hypothesized that, especially in tropical environments, plant-microbe interactions may lead to horizontal gene transfers (HGT) or genetic recombinations, from the plant to its endophytic counterpart or vice versa, that subsquently, lead to “novel” endophytes capable of accumulating certain metabolites specific to the host plants themselves. Although only certain specific novel endophytes are capable of producing certain host-specific compounds and host-microbe coevolution procedures are still unknown, these methods may present a new perspective for the production of natural products and have several advantages such as low cost, the use of renewable resources and finally reproducible results by use of fermentation technologies. In this framework, the possible microbial mechanism of hypericin and emodin biosynthesis was studied in axenic submerged culture conditions in the endophytic fungus *Thiela*v*ia subthermophila*, isolated from *H. perforatum* [16,17]. Results showed that neither emodin anthrone nor protohypericin could be detected at any stage of fermentation, irrespective of either spiking or illumination conditions. Furthermore, the Hyp-1 gene was absent in *T. subthermophila*, indicating that the biosynthetic pathway in the endophytic fungus might be different and/or governed by a different molecular mechanism than the host plant or host cell suspension cultures.

## 2. Biosynthesis

**Figure 3.**
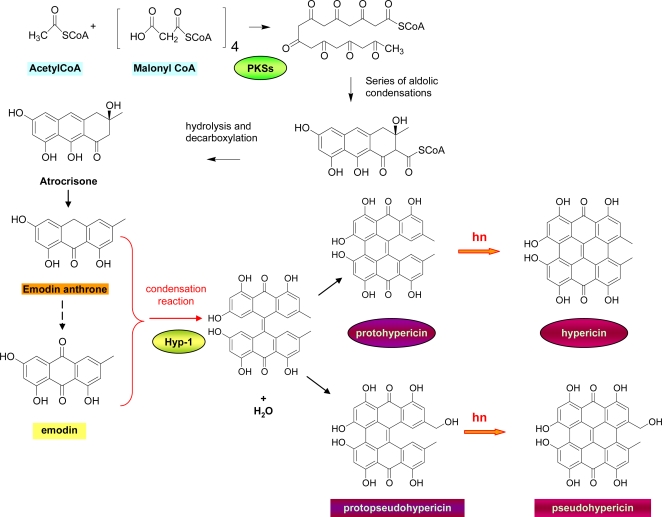
Biosynthesis of hypericin. Oxidation towards the pseudohypericin pathway is presumed to occur after the condensation reaction.

The biosynthesis of hypericin and pseudohypericin, is presumed to follow the polyketide pathway [1], according to which, in a first step one molecule of acetyl CoA condensates with seven molecules of malonyl CoA to form an octaketide chain that subsequently undergoes cyclizations and decarboxylation, leading to the formation of emodin anthrone (Figure 3).

Emodin anthrone is considered to be the precursor of hypericin. As it has been suggested [8,18], emodin anthrone is oxidized to emodin, probably by the enzyme emodinanthrone-oxygenase and then through a condensation reaction yields a dianthrone. Successive oxidations lead to the formation of protohypericin, which upon irradiation with visible light yields hypericin. Oxidation of the methyl group of protohypericin is presumed to lead to protopseudohypercin, which is similarly transformed to pseudohypercin under visible light. This biosynthetic root is generally accepted today, even though further investigations that would prove it are necessary, and most importantly, the polyketide synthases that would play a crucial role have not yet been characterised. Very recently, however, two cDNAs encoding for polyketide synthases (PKSs), designated as HpPKS1 and HpPKS2, were isolated from *H. perforatum* [19]. The HpPKS1 expression was highest in flower buds and lowest in root tissues and was found to correlate with the concentrations of hyperforin and adhyperforin in *H. perforatum* tissues, while the expression of HpPKS2 showed correlation with the concentrations of hypericins and pseudohypericins and was found to be high in flower buds and leaf margins and low in leaf interior parts, stems and roots.

Further studies by Bais *et al*. [20] revealed the presence of an enzyme, Hyp-1, from darkgrown *H. perforatum* cell cultures, that specifically catalyzes the direct conversion of the anthraquinone emodin to hypericin *in vitro*. It is hypothesized that Hyp-1 catalyzes an initial condensation reaction between emodin and emodin anthrone followed by a dehydration to form emodin dianthrone. Emodin dianthrone may eventually undergo phenolic oxidation to protohypericin, either by photoactivation or by Hyp-1 itself. Kosuth, *et al*. [21] studied the expression of the Hyp-1 gene in different organs of *H. perforatum* seedlings in early stages of development. Surprisingly, the highest level of expression was found in roots. This fact indicates that the sites of biogenesis and accumulation of hypericin in the *Hypericum* plants are independent of the expression of the Hyp-1 gene.

## 3. Properties

Hypericin has been extensively studied for its physicochemical properties, little is known however, for pseudohypericin. Hypericin is not planar, as indicated by X-ray diffraction studies [22]. The side chains of the aromatic skeletons repel each other and prevent the molecule from acquiring a planar conformation.

There are 16 theoretical tautomers of hypericin [23], but the more stable one is the 7,14-dioxoisomer. In concentrated hypericin solutions, the 1,6-dioxo isomer converts slowly into the 7,14-dioxoisomer. Dilution of the solution or addition of pyridine DMSO, or other polar compounds accelerates the reaction. The 1,6-dioxo-tautomer is stabilized in the solid phase and concentrated solutions by intermolecular hydrogen bonds. Salts of hypericin with alkali metals retain the structure of the 7,14-dioxo-tautomer both in solutions and crystalline state [24].

Hypericin has the affinity for forming associates. It dissolves monomolecularly in common polar solvents up to concentrations of 10^−3^ mol/L. Under certain conditions (*i.e*., in the presence of water), however, it forms homoassociates. The form of the homoassociates depends on the tautomeric state of hypericin. Hydrophobic contacts of the aromatic core are responsible for the 7,14-dioxo tautomer, whereas hydrogen bonding is responsible for the formation of the homoassociates of the 1,6-dioxo tautomer. These homoassociates influence considerably the absorption spectra of hypericin and this should be considered in the case of HPLC-DAD analyses. For example, the homoassociates of the 1,6-dioxo tautomer show high extinction coefficients and narrow absorption bands [24,25] than those of the 7,14-dioxo tautomer. Falk and Meyer [25] reported that the extinction coefficient of hypericin in water is one fourth of that in pure dimethylsulfoxide.

Other factors that influence the absorption spectra of hypericin, are solvent, the pH, its ionization [26], its tautomeric and conformational state [27]. Transformation from one tautomer into another only occurs in solution. Dilution, rise in temperature, addition of pyridine and dimethylsulfoxide disturb the hydrogen bonds by dissolving the hypericin molecules, and therefore enhance the dissociation of the homoassociates.

The proximity of acidic and basic functional groups on hypericin allows the formation of several hydrogen bonds which play a crucial role in the molecular recognition and specificity of its interactions with molecular targets. X-ray data indicated that, deprotonated hypericin forms five short intramolecular hydrogen bonds which influence the tautomeric equilibria and determine its acido-basic properties. Hypericin tends to dissociate one proton in polar solvents to give birth to hypericin anion [28]. This, anion influences the photosensitive features of hypericin. Furthermore, it has been suggested that an intramolecular proton transfer takes place and that the reaction is coupled to intramolecular conformational changes (excited-state tautomerization) [29,30].

The naphthodianthrones are poorely soluble in water [31], with pseudohypericin being more soluble in polar solvents due to the presence of the extra hydroxymethyl unit. It has been suggested that procyanidins could be responsible for the solubilization of naphthodianthrones in aqueous solutions [32] by forming complexes. Potassium salts which increase the solubility and the extractability from the plant have also been described [26]. Binding to human serum albumin helps hypericin to solubilize in aqueous physiological solutions, as it leads in the dissociation of the aggregates usually formed in the aqueous phase into the monomeric form of hypericin [33]. Standard solutions of naphthodianthrones in methanol:pyridine (99:1, v/v) have been proposed as a means to dilute completely naphthodianthrones. The improved solubility upon the addition of pyridine is explained by the formation of naphthodianhtrone-pyridine complexes. Solutions have to be kept in the dark [32] to avoid photodegradation. Pseudohypericin degradation however, is slightly accelerated by the addition of pyridine.

## 4. Stability

Both substances are labile, especially under light. They seem to be more stable in St. John’s Wort extracts up to 48 h [34] but the stability of *H. perforatum* extracts is of no interest in the present review. Maisenbacher [35] studied the photodegradation of hypericin in acetone using a day lamp for a duration of 40 h. The samples were monitored with NMR and the authors concluded that hypericin degraded to lipophilic derivatives. Wirz *et al*. [36], studied the stability of standard solutions of hypericin and pseudohypericin. All solutions were stable at −20 °C in darkness over the investigated period of 140 days. Higher temperatures, light and the presence of pyridine accelerated the degradation of pseudohypericin, light exposure being the most aggressive factor. Hypericin showed higher stability, than pseudohypericin in light. The instability in the presence of light was more pronounced in the extract solution both for hypericin and pseudohypericin. Under all the other storage conditions, the stability of pseudohypericin was better in the extract solutions than in the standard solutions. pH is another factor that causes degradation of the naphthodianthrones. Acidic conditions caused decomposition of hypericin and pseudohypericin in extracts of *H. perforatum*, both under light and dark conditions [37]. Similarly, alkaline conditions caused a rapid degradation of pseudohypericin in standard solution or in *Hypericum* extracts [38,39]. According to our experience [40], standard solutions of pure hypericin and pseudohypericin presented a loss of 20% after four days at room conditions (temperature and light). These results highlight the need to carry out all isolations and handling of the compounds as fast as possible.

## 5. Extraction, Isolation and Synthesis of Hypericin and Pseudohypericin

### 5.1. Extraction and Isolation

Several extraction methods of naphthodianthrones from Hyperici herba have been described, including maceration, sonication, Soxhlet extraction, supercritical fluid extraction and pressurized liquid extraction. Due to the very low content of naphthodianthrones in *H. perforatum* the extraction processes are of high cost and require multiple cycles and fast handling of the material, since hypericins are labile substances. The European Pharmacopaeia uses THF for the extraction.

The extraction methods that have been applied use hydroalcoholic mixtures in different ratios. There are many contradictions in the literature concerning the solvent selection. In general, lower percentage in water favors the extraction of hypericin compared to pseudohypericin. Ang *et al.* combined methanol 100% with sonication to quantitatively extract the naphthodianthrones [37,41], while Draves *et al*. [42] reported the combination of ethanol 100% with sonication. The extraction was found to be rapid and to recover more than 95% of both pseudohypericin and hypericin following 1 h of sonication.

Williams *et al*. compared different methods using a variety of solvent, temperature and time conditions [11]. In particular, they performed (i) sonication with methanol for 120 min at 60 °C, (ii) Soxhlet extractions over five time intervals at five different extraction times: 52 min, 140 min (six cycles), 8, 24, and 48 h. and (iii) pressurized liquid extraction using methanol, tetrahydrofuran, acetone, methylene chloride, and hexane at room temperature. PLEs were also carried out at several elevated temperatures, 60, 100, 150, and 200 °C, using methanol. They concluded that the highest levels of polar and nonpolar constituents were achieved through Soxhlet extraction of botanical samples over a 24 h period. In general, more complete extractions resulted with longer extraction times and multiple solvent contacts. Higher yields of polar constituents were achieved with tetrahydrofuran, whereas less polar constituents such as hypericin and pseudohypericin were more completely extracted with acetone.

Similar results reported recently Smelcerovic *et al*. [43], who compared direct sonication with conventional maceration, indirect sonication, Soxhlet extraction, and accelerated solvent extraction (ASE). Six active compounds were considered, among them hypericin and pseudohypericin. Direct sonication gave the best results. Conventional maceration gave the lowest amount of analyzed active compounds. Soxhlet extraction gave better results than ASE or indirect sonication.

Another interesting study was reported by Benthin *et al*. [44]. They developed an extraction process of multiple steps and compared it with the method used in the German Drug Code (DAC). After a preliminary removal of undesirable lipophilic constituents by one extraction cycle with CH_2_Cl_2_ at 100 °C, dianthrons were extracted by three cycles of 5 min each with MeOH at temperatures ranging from 50 to 100 °C. Extraction cycles 2 and 3 afforded further hypericin, but with significantly decreasing yields. The yield of total dianthrons was significantly higher in the PLE than in the extracts obtained by the German Drug Code method. In the contrary, supercritical extraction is unsuitable, as it leads to the isolation of the unpolar hyperforins [45].

Due to the very low content of hypericin and pseudohypericin in *Hypericum* sp. the isolation of the naphthodianthrones is a complicated procedure, including many different separation steps. In the past various methods of isolation have been developed which mainly consist of HPLC analyses on RP-C_18_ columns [37,46]. In other cases isolation protocols include the application of successive column chromatographies over silica [47,48], polyamide [49] or Sephadex LH-20 [50,51] which render the whole procedure labour-intensive and time consuming and as a consequence very expensive. A very interesting work on different separation methods of hypericin and pseudohypericin from *H. perforatum* was published by Smelcerovic *et al*. 2002 [52]. Flash chromatography, high speed counter-current chromatography, XAD solid phase extraction and Sephadex LH-20 column chromatography were tested, with the latter being proved to be the most convenient method for the separation of this type of constituents. Separation by Sephadex LH-20 afforded a fraction containing 33.7% hypericin and 40% pseudohypericin. However, a final purification by preparative HPLC was also needed. An elaborated but complicated isolation scheme was described by Wirz [53]. In brief, the method consisted of an initial liquid liquid partition with the system hexane-toluene-H_2_O-EtOAc-HCOOH (75:225:135:120:15, v/v), the upper level was collected and evaporated, redissolved in methanol and stored at 8 °C. A precipitated was formed which was subjected to high-speed counter current chromatography (HSCCC) using the solvent system toluene - acetonitrile - water - ethanol absolute (3:4:3:2, v/v) to yield pure hypericin and pseudohypericin. The first step was repeated four times in order to obtain a sufficient amount of pure naphthodianthrones.

Recently, Karioti *et al*. [40], reported an isolation scheme which combined partition and size exclusion chromatography using different Sephadex gels. According to this method the *H. perforatum* dried methanolic extract was redissolved in aqueous methanol (20%) and extracted initially with hexane in order to remove unwanted lipids and chlorophylls which absorb in the same UV range as naphthodianthrones. After partition with Et_2_O and EtOAc, the extracts were combined and further purified by successive polar (MeOH 70%) and lipophilic (EtOAc:MeOH 70:30) Sephadex LH-20 to yield high purity naphthodianthrones. In a last step a Sephadex LH-60 was applied to separate hypericin from pseudohypericin. Among the advantages of this method were the simplicity and the use of open short columns (and therefore fast). Scaling up of the procedure to higher amounts up to 10 g or 35 g was feasible and with reproducible results.

### 5.2. Synthesis

Hypericins’ syntheses could be divided into two groups: (i) synthesis of the original constituents with the lowest possible cost in order to provide higher amounts of these rare compounds and (ii) synthesis of derivatives, the so-called, second-generation agents, characterized by redshifted absorption spectra, excitable at wavelengths with enhanced penetration depth and improved physicochemical properties, such as solubility, an enhanced ability to generate singlet oxygen and/or reactive oxygen species, bioavailability, or targeting of specific cellular sites.

#### 5.2.1. Synthesis of hypericin and pseudohypericin

The chemical synthesis of hypericin follows the pattern of the proposed biogenesis. The first synthesis was achieved by Brockmann [54,55]. Later, Falk and coworkers proposed a semisynthetic route starting from the easily available anthraquinone precursor emodin [56] and, in parallel, they developed a convenient synthesis of emodin [57]. Recently, the same group proposed an improved method by employing microwave technology for the preparation of hypericin in higher yields [58]. With this methodology dimerization of identical “halves” (anthrones) leads to symmetrical derivatives, while, most importantly, by using or mixed “halves” (anthrone and quinone) *via* the corresponding protohypericin. The same method was applied with success for the preparation of hypericin derivatives ans most importantly it can be used for the production of unsymmetrical hypericin derivatives. Main advantages of the application of microwave assisted solid phase synthesis for the preparation hypericins, apart from the higher yield, are the absence of high boiling solvent and decreased reaction time. A six step synthesis of hypericin *via* emodin anthrone starting from the simple 1,4-benzoquinone was reported by Motoyoshiya *et al*. [59].

#### 5.2.2. Synthesis of analogues with improved physicochemical properties, solubility and targeting of specific cellular sites

A great variety of synthetic hypericin derivatives has been lately designed aiming to produce molecules with improved physicochemical properties, enhanced solubility, interaction with DNA, accumulation in particular tissues and finally acting as imaging agents. Some of the reports provide data on the efficacy of such products. A simple method to determine the phtochemical behaviour of the synthetized compound is to irradiate the hypericin derivatives in the presence of sodium bilirubinate IXa. With this method the photosensitized production of singlet oxygen and/or reactive oxygen species from molecular oxygen in aerated solutions is determined by monitoring the oxidative destruction of bilirubin IXa.

Falk and co-workers, renowned for their research in hypericin synthesis and the elucidation of the physicochemical properties of this molecule, developed a new class of modified hypericin derivatives, which contained extra heterocyclic rings such as benzothiazolyl, benzoxazolyl [60] and benzothiazole [61]. Basic concept of these syntheses was that the methyl groups of the hypericin moiety served as “anchors” for the substitution by the extra chromophore units. In this way, the photophysical properties of the hypericin chromophore remained more or less untouched. In parallel, the same team developed a group of amino functionalized hypericin derivatives [62] with excellent overall yields and enhanced solubility.

The observation that nitrogen containing heterocyclically substituted derivatives presented a pronounced bathochromically shifted absorption spectra, led to the design of a new series of derivatives, where the hypericin chromophore was fused with nitrogen containing heterocyclic rings: phthalazines, phthalazinones and pyridazinones [63]. Generally, the improved photochemical properties of these derivatives are attributed to the nature of the fused aromatic heterocycle which allows an efficient delocalization of the π-electrons of the phenanthroperylenequinone moiety.

Hypericin derivatives containing dicyclohexylurea moieties have been also synthetized. They showed excellent production of oxidizing species comparable to hypericin. [64]. The authors however, do not mention how the insertion of the sulfur containing unit could modify the interaction with cell components.

Hybridization products of hypericin with porphyrin have been described. The concept of this synthesis was to combine the intense long-wavelength absorption of hypericin with the very weakly absorbing, but highly tumor-targeting properties of porphyrins [65].

In an attempt to create hypericin derivatives potentially interacting with the complementary nucleobases of DNA or RNA by Watson-Crick pairing, or intercalation, or with other constituents of the cell nucleus, hybridization of hypericin with nucleobases was performed. Although no specific interaction with DNA or Watson-Crick base pairing with poly(20-deoxyadenylic acid) could be detected, the products accumulated in the nucleus of prostatic cancer cells better than hypericin. [66].

Crnolatac *et al*. [67], synthesized a series of more lipophilic hypericins *i.e*., hexyl-, octyl-, decyland dodecylamides of hypericin acid. *In vitro* cellular uptake and photo-induced antiproliferative effects of the compounds were evaluated, using the human moderately differentiated non-invasive papillary transitional carcinoma RT-112 cell line, whereas the more lipophilic amides were taken up limitedly, the hexylamide accumulated approximately as well as hypericin itself.

The synthesis of radioiodine labeled hypericin was also reported as a malignant glioma imaging agent. [68]. In all glioma cell lines, 2-[123I]iodohypericin uptake was increased in a time dependent manner and an accumulation of 2-[124I]iodohypericin was observed in C6 glioma bearing nude mouse. The results suggested that radioiodine labeled hypericin could visualize a PKC overexpressed malignant glioma. In contrast, a 99mTc-labeled hypericin derivative did not show preferential uptake in necrotic tissue [69] and thus considered as not suitable for imaging necrosis.

A second generation carbohydrate-linked hypericinic photosensitizing agent was synthetized with enhanced solubility [70]. Its photochemical properties resulted to be better than those of hypericin, while it showed binding-interactions with DNA.

Finally, unusual cationic hypericin derivatives were synthetized and further assayed for their photobactericidal activity against *Propionibacterium acnes* [71]. The quaternary *N,N*,*N*,-trimethylanilinium derivative of hypericin displayed a pronounced photodynamic inactivation of the bacteria at low incubation concentrations (<100 nm) and a short incubation time (1 h) after illumination.

## 6. Photodynamic Therapy (PDT) and Cancer

Hypericin has been extensively studied for its application in the photodynamic therapy. Photodynamic therapy (PDT) involves the administration of a tumor-localizing nontoxic drug known as photosensitizer, systemically, locally, or topically. The photosensitizer should accumulate in the tumor. After an incubation period follows illumination of the tissue or tumor with visible light (usually long wavelength red light) in the presence of oxygen, during which the photosensitizer (PS) is activated generating reactive oxygen species toxic to the tumor cells and thus leads to tumor death and tissue destruction.

**Figure 4.**
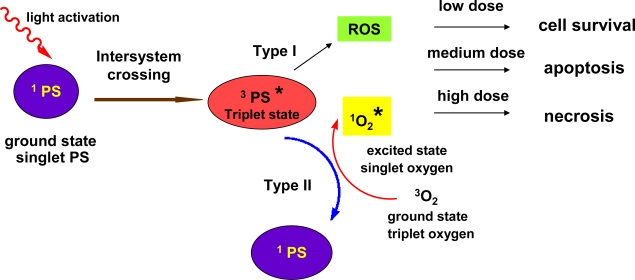
Mechanism of photoactivation of hypericin and induced damages.

The use of PDT as a cancer therapy is particularly attractive because of its selectivity. This is better understood once the mechanism of PDT is considered. The photophysical processes that take place during PDT are very well explained in the review article by Castano *et al*. [72]. In brief, the PS absorbs light and is boosted from the ground state or singlet state (low energy at which the electrons occupy opposite spins) to the first excited singlet state. The excited singlet state PS may also undergo the process known as intersystem crossing whereby the spin of the excited electron inverts to form the so called excited triplet-state (electron spins parallel). The PS excited triplet can undergo two kinds of reactions (Figure 4): Type 1 reaction, during which it can react directly with a substrate, such as the cell membrane or a molecule, and Type 2 reaction, where the triplet PS can transfer its energy directly to molecular oxygen (itself a triplet in the ground state), to form excited state singlet oxygen. Both Type 1 and Type 2 reactions generate further reactions that have as a result the formation of ROS which are toxic to several cell structures and macromolecules (DNA, lipids, enzymes). Only molecules and structures that are proximal to the area of ROS production (areas of PS localization) are directly affected by PDT. Due to the fact that the PS localizes in the malignant tissue the light is also spatially focused on the tumor.

Three distinct (but possibly interrelated) mechanisms are involved in the observed shrinkage of tumors during PDT: (i) generated ROS kill tumor cells directly by apoptosis and/or necrosis; (ii) damages of the tumor-associated vasculature, lead to tissue deprivation of oxygen and nutrients and consequent tumor infarction and (iii) activation of immune responses against tumor cells [72,73].

Naphthodianthrones have a strong ability to generate singlet oxygen and other reactive oxygen species upon irradiation. Especially hypericin is one of the most powerful photosensitizers in nature. Vandenbogaerde *et al*. [74], studied comparatively the photocytotoxic effect of hypericin and pseudohypericin. Despite the structural resemblance pseudohypericin appears to be less effective than hypericin, probably due to decreased uptake of the former by the cells associated to irreversible interaction between pseudohypericin and serum constituents. Most importantly, this strong binding to foetal calf serum resulted in decreased fluoroscence. The antitumoral activity of hypericin is oxygen dependent suggesting that both Type I and Type II mechanisms of photodynamic action are involved. [75,76]. It has been suggested that the Type II mechanism is the one to play a major role in the biological photoactivity of hypericin. However, newer evidence show that there may be an additional mechanism of photoactivation involving an intramolecular proton (or hydrogen) transfer, as discussed previously in Section 3.

In the last years there is a continuously growing evidence of the effectiveness of hypericin in PDT. Most reports concern *in vitro* studies on a variety of cell lines. Cells are firstly incubated with hypericin, irradiated and finally assessed by MTT assay for cell viability. Since this issue is analyzed extensively in previous review articles [77,78] in this report we will focus on newer data. In Table 1 are summarized newer published reports. In all the investigations listed light activation was mandatory for the expression of the cytotoxic activity of hypericin. Of more interest are *in vitro* tests which gave positive results against childhood rhabdomyosarcoma and pediatric epithelial liver tumors.

The phototoxic effect of UVA (400–315 nm)-activated hypericin in human pigmented versus unpigmented melanomas was also studied with *in vitro* assays [79]. It was reported that 3 μM of activated hypericin induced a necrotic mode of cell death in pigmented melanoma cells and melanocytes and an apoptotic mode of cell death in non-pigmented melanoma cells and keratinocytes. This difference in cell death in pigmented melanoma cells and melanocytes was attributed to the presence of melanin-containing melanosomes which under the strong oxidative effect of reactive oxygen species released toxic melanin precursors (of an indolic and phenolic nature) which led to cell death. In contrast, non-pigmented melanoma cells and keratinocytes died by apoptosis related to a mitochondrial caspase-dependent mechanism. The same authors reported however [80], that exposure to hypericin induced initially a cytoprotective (autophagic) response from both cell lines, but in the end the cells succumbed with different susceptibilities to the hypericin induced stress. In addition, pigmented cells accumulated a large amount of glycogen in their cytoplasm which might be associated with inhibition of GSK3, which in case of melanoma has been linked to different modes of cell death.

Some interesting findings concern the combination of hypericin with other agents in order to maximize its efficiency. A high sensitivity combination scheme can be also exploited in conventional cytological diagnosis or even in ex vivo fluorescence cytology [81]. Schneider-Yin *et al*. [82], reported that treatment of endometrial cancer cells with both aminolevulinic acid and hypericin followed by irradiation with white light induced a significantly higher phototoxicity. In another study, during which colon cancer cells HT-29 were pre-treated with the specific 5-lipoxygenase inhibitor MK-886, it was evidenced that cancer cells became more susceptible to photodynamic therapy (PDT) with hypericin and the combination induced cell cycle arrest and stimulated onset of apoptosis [83]. It was further presumed that pre-treatment with MK-886 modulated distribution of ROS production in mutual combination with PDT [84] and that apoptosis was accomplished preferentially through the mitochondrial pathway although caspase-8 activation was also noticed. However, recent findings show that high levels of arachidonic acid, a cyclooxygenase-2 and 5-lipoxygenase substrate, could act as death messenger and further induce apoptotic signals (For more details see Section 6.1).

### 6.1. Mechanisms Linked to Apoptotic/Necrotic or Cell Survival Processes

**Figure 5.**
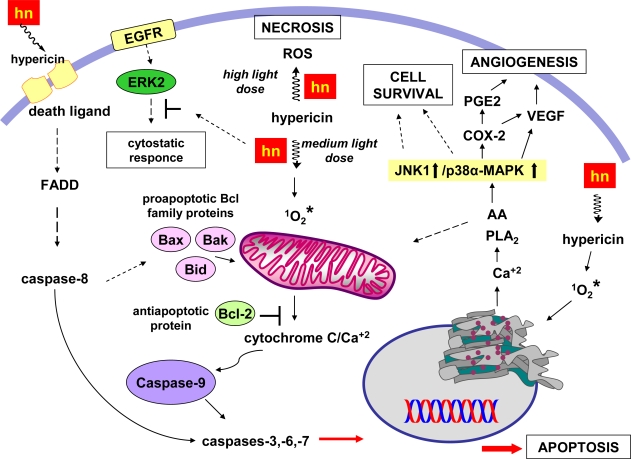
Some of the multiple and interrelating signaling pathways induced during PDT with hypericin.

Many efforts have been made in order to comprehend the cellular processes implicated in hypericin PDT. The subcellular localization of hypericin, the type of the tissue/cell and the light dose are some of the factors that influence the fate of the cancer cells: necrosis, apoptosis, or survival. Different cellular mechanisms are activated in each case, but most of the times co-evolve in different proportions and interrelate [85] (Figure 5). The photocytotoxic pathways that have been identified include inhibition of protein kinases (C, A), protein tyrosine kinases and other growth factor-stimulated protein kinases [77], membrane lipid peroxidation [86], downregulation of matrix metalloproteinase-1 [87], increase of superoxide dismutase activity, decrease of cellular glutathione level [88]. Mitochondrial apoptotic pathways are also responsible, such as inhibition of mitochondria outer membrane bound hexokinase, or activation of the caspase signalling system and release of cytochrome c into the cytosol. Mitochondrial damage has been identified as one of the main events during PDT with hypericin [89]. In contrast, soon after irradiation, JNK1 and MAPK (Mitogen-Activated Protein Kinase) pathways are activated independently of the caspace activation leading to an increased cellular resistance against hypericin induced apoptosis [78]. Other cellular mechanisms that may contribute to cell resistance during PDT with hypericin include intracellular redox enzymatic systems with free radical scavenging capabilities such as the glutathione S-transferase, glutathione reductase and others.

Necrosis was found to be the principal procedure of cell death in colon adenocarcinoma HT-29 cells [90]. This occurred irrespectively of different PDT doses and the absence of anti-apoptotic Bcl-2 expression. Introduction of Bcl-2 into HT-29 cells caused caspase-3 activation, changed the Bcl-X_L_ expression pattern, increased the apoptosis ratio with no effect on overall toxicity, and supported arrest in the G_2_/M-phase of cell cycle. Since it is known that Bcl-2 suppression in HT-29 is reversible and linked to the over-expression of mutated p53 and also considering our data, we suggest that the mutation in p53 and events linked to this feature may play a role in cell death signalling in HT-29 colon cancer cells.

Similarly, in an *in vitro* study on human epidermoid carcinoma cells (A431) [91], a transition from apoptosis to necrotic cell death was observed at higher light doses. Intermediate ligh doses of 1.44 J/cm^2^ induced apoptosis. Time resolved analysis of the apoptotic processes showed a significant activation of caspase-2, -3, -6, and -9, and most importantly, rapid reduction of the mitochondrial membrane potential which clearly proved mitochondrial involvement in the apoptotic process. Analysis of the energetic characteristics of hypericin-PDT induced apoptosis revealed that the levels of intracellular ATP remained at control level for up to 6 h post irradiation. This fact suggested that the energetic supply for the ATP dependent steps during apoptotic cell death is independent of mitochondrial ATP synthesis. Upregulation of glycolysis may have served as a compensating mechanism of energy supply.

1. high dose of light leads to necrosis.
2. medium light doses activate different apoptotic pathways: (2a) activation of caspase -8 and final activation of the caspases -3, -6, -7; (2b) mitochondrial release of cytochrome C, accompanied by mitochondrial Ca^2+^ release leads to activation of the caspases -3, -6, -7 *via* activation of caspace -9. Bax/Bid proapoptotic proteins enhance the cytochrome C release, whereas the antiapoprotic Bcl-2 inhibits the cytochrome C reflux; (2c) Ca^2+^ release from the endoplasmic reticulum activates cytochrome C release from mitochondria.
3. inhibition of the ERKs induces cytostatic responces.
4. low light doses favor the MAPKs pathway leading to cell survival: activation of MAPKs JNK1 and p38α pathways leads to cell survival and angiogenesis.

Apoptosis events seem also to be related with a raise of the intracellular Ca^2+^ levels which occurs *via* influx of Ca^2+^ through ion channels, release of Ca^2+^ from the endoplasmic reticulum or mitochondria or ion exchange mechanisms [85]. In an *in vitro* study on human prostate carcinoma cells mitochondrial aconitase, an enzyme exquisitely sensitive to oxidation, revealed a dose correlated loss of activity immediately after hypericin photoactivation, suggesting mitochondrial impairment. Combination with ionomycin, which modulates both internal Ca^2+^ stores and external Ca^2+^ transport, profoundly enhanced photocytotoxicity [92].

In an *in vitro* analysis of the local and temporal changes of the Ca^2+^ concentration after hypericin-PDT in human U373 glioblastoma cells [92] it was shown that low light doses (under 1 J/cm^2^) induced Ca^2+^ oscillations, which might be explained as Ca^2+^ transport between mitochondria and endoplasmic reticulum. On the contrary, at higher light doses (over 3 J/cm^2^), Ca^2+^ overload and subsequent Ca^2+^ release into the cytoplasm were observed. Furthermore, after incubation of cells with hypericin and the mitochondrial marker MitoTracker green, a colocalization of the two dyes was observed in mitochondria, suggesting that these cellular organelles consist a major target of the hypericin phototoxicity. Authors proposed that the damage of mitochondrial ion pumps caused by reactive oxygen species could be responsible for the temporal changes in the cytosolic Ca^2+^ concentrations observed.

The role of endoplasmic reticulum in the intrinsic apoptosis and its relation to the Ca^2+^ homeostasis was the subject of another study by Buytaert, *et al*. [94]. In general, the antiapoptotic Bcl-2 protein protects cells from stress-induced apoptosis by reducing the resting endoplasmic reticulum Ca^2+^, whereas proapoptotic Bax and Bak proteins are required to maintain endoplasmic reticulum Ca^2+^ pool necessary for the induction of apoptosis by Ca^2+^ mobilizing stress signals [95]. Buytaert, *et al*. proposed that during PDT with hypericin occurs loss of the native SERCA2 protein levels and consequent endoplasmic reticulum Ca^2+^ depletion which causes disruption of Ca^2+^ homeostasis and cell death. Apoptosis was rapidly initiated after endoplasmic reticulum Ca^2+^ depletion, while the multidomain Bax/Bak proteins are strictly required for fast effector caspase activation and induction of intrinsic apoptosis. It was also observed that in the absence of Bax and Bak proteins (Bax–/–Bak–/–double-knockout cells), cells are protected from apoptosis but undergo autophagy-associated cell death [96]. It was concluded that after endoplasmic reticulum photodamage and disruption of Ca^2+^ homeostasis, Bax and Bak proteins model PDT-mediated cell killing, which is executed through apoptosis in their presence or *via* an autophagic pathway in their absence.

Dua *et al.* [97] reported the down-regulation of matrix metalloproteinase-9 following hypericin-PDT in well-differentiated human nasopharyngeal cancer cells infected by the Epstein Barr virus. Epstein Barr is strongly related to the promotion of nasopharyngeal cancer and also with the expression of MMP-9. The authors initially confirmed the presence in the HK1 NPC genome of the Epstein Barr virus encoded RNA 1 (EBER1) transcript. It was postulated that the observed down-regulation of matrix metalloproteinase-9 was due to inhibition of granulocytemacrophage colonystimulating factor (GM-CSF) production which resulted in decreased binding activity of NF-kB and AP-1 transcription factors.

MAPKs are found in all eukaryotic organisms and although they can regulate cytoplasmic targets, their ultimate role is to transmit extracellular signals to the nucleus where the transcription of specific genes is induced by phosphorylation and activation of transcription factors. Three distinct MAPK pathways have been characterized in mammalian cells: the extracellular signal-regulated kinase (ERK) cascade, the c-Jun N-terminal kinase (JNK; also called the stress-activated protein kinase, SAPK) cascade, and the p38 MAP kinase cascade [98].

The role of PDT activated MAPKs in cell death regulation has not been fully elucidated. The ERK pathway (ERK1/2) is associated with cell survival, is activated by growth factors and induces cell proliferation [99,100]. The JNK and p38 MAPK activation cascades are activated mainly in response to a variety of extracellular and intracellular stress-stimuli such as oxidative stress, inflammatory cytokines and UV insult which is related to the activation of the cell death signalling pathways [101,102]. Four isoforms of MAPK superfamily, p38-α, -β, -γ and -δ, exist, commonly activated by cellular stress, proinflammatory cytokines and growth factors [103].

Based in newer scientific evidence, hypericin-PDT activates rescuing responses, chiefly governed by the activation of P38 MAPK: JNK1 and p38 MAPK are activated, while ERK2 is irreversibly inhibited [104,105]. This activation is independent of the caspase activation cascade and functionally signals to a survival response during PDT with hypericin, without however, rescuing cells from death. Most likely they delay the apoptotic process by inducing the transcription of survival-promoting genes [106] until a critical threshold of damage finally commits the cells to apoptosis [104].

Chan and coworkers [107] have investigated the role of MAPKs in HY-PDT-induced apoptosis of nasopharyngeal HK-1 NPC cancer cells using both chemical inhibitors and small interfering RNA (siRNA). Results showed that mitochondrial (Bax) translocation and formation of Bax channel, as well as proteolytic cleavage of procaspase-9 and -3 in HK-1 cells took place. The observed increase in phosphorylation of p38 MAPKs and c-Jun N-terminal kinase 1/2 (JNK1/2) was inhibited by the use of the singlet oxygen scavenger L-histidine, showing that MAPKs pathways are activated *via* the induction of oxidative stress in HK-1 cells, whereas blocking the expression p38a MAPK (p38a) and p38b MAPK (p38b) isoforms of the MAPKs enhanced the HY-PDT-induced apoptosis of HK-1.

Buytaert and coworkers [108] studied the gene expression following hypericin PDT in bladder cancer cells. Results demonstrated that PDT strongly affects various metabolic processes, stress-induced cell death, autophagy, proliferation, inflammation and carcinogenesis. Analysis of PDT-treated cells after p38-MAPK inhibition or silencing unraveled that the induction of an important subset of differentially expressed genes regulating growth and invasion, as well as adaptive mechanisms against oxidative stress, is governed by this stress-activated kinase. Moreover, p38-MAPK inhibition blocked autonomous regrowth and migration of cancer cells escaping PDT-induced cell death. Authors concluded that hypericin-PDT pinpoints a coordinated induction of a cluster of genes involved in the unfolded protein response pathway after endoplasmic reticulum stress and in antioxidant response. This analysis identifies new molecular effectors of the cancer cell response to PDT opening attractive avenues to improve the therapeutic efficacy of hypericin-based PDT of bladder cancer.

From a recent study, activation of p38a MAPK appears to be crucial for the induction of cycloxygenase-2 (COX-2) expression and PGE2 synthesis in the hypericin PDT-treated cells [109]. This finding is very important, since the p38a MAPK mediated COX-2 expression may facilitate the growth of cells endowed with resistance to PDT and with the potential to progress to malignancy. It has been evidenced that hypericin PDT leads to a rapid rise in the levels of cytosolic calcium which generates the accumulation of arachidonic acid *via* phospholipase A_2_ activation (PLA2) (Figure 5). Arachidonic acid seems to act as a death messenger for the cell and its depletion by overexpressed cyclooxygenase-2 leads to further synthesis of prostaglandin PGE2 and upregulation of vascular endothelial growth factors (VEGF), which in turn favors tumor neoangiogenesis, thereby supporting local tumor progression and metastatic spreading. Hendrickx and coworkers [109] demostrated that inhibition of p38α MAPK blocked the release of vascular endothelial growth factor and suppressed tumor-promoted endothelial cell migration. Hence, targeted inhibition of p38α MAPK could be therapeutically beneficial to PDT, since it would prevent COX-2 expression, the inducible release of growth and angiogenic factors by the cancer cells, and cause an increase in the levels of free arachidonic acid which promotes apoptosis.

Hence, the specific targeting of p38a MAPK surpasses the effect of COX-2 enzymatic inhibition as it possibly targets additional mediators of the survival and angiogenic switch in cancer cells. In fact, emerging evidence indicates that besides regulating COX-2 expression, the activationof p38 MAPK can critically contribute to the process of carcinogenesis by regulating the turnover of labile metastatic gene transcripts, including matrix metalloproteinases and urokinase-type plasminogen activator, as well as by up-regulating genes involved in the adaptive response to oxidative stress. In addition, it is possible that the p38MAPK pathway regulates the activity of other AA-metabolizing enzymes besides COX-2, such as 5-lipoxygenase, as the simultaneous inhibition of these AA-converting enzymes is required to increase the susceptibility of the T24 cells to PDT-induced cell death. Since eicosanoids produced by the activation of lipoxygenases have been reported to stimulate VEGF synthesis, this could also explain why blockage of p38 MAPK is more effective in suppressing the VEGF release than the unique inhibition of COX-2. Moreover, recent studies have indicated that some COX-2 growth-promoting functions are independent of the COX-2 enzymatic activity. Hence, the inhibition of p38 MAPK, resulting in the shut down of COX-2 expression, appears more promising as new treatment strategy in our paradigm than the use of COX-2 inhibitors, since it may encompass all COX-2-dependent phenotypes, including those merely mediated by its elevated expression.

Taken together, these results encourage further in vivo studies to assess the potential antiangiogenic and pro-apoptotic role of the combined pharmacological inhibition of p38 MAPK in hypericin-based PDT of tumors.

### 6.2. Modulation by Redox Cellular Systems

The hypericin-mediated photodynamic effects are often modulated by cellular antioxidant defense mechanisms. Under extreme stress conditions cells activate their antioxidant defense mechanisms against reactive oxygen species in order to cope with the initial radicals produced, but also the resultant and potentially more toxic products of the catalyzed and spontaneous reduction reactions. Among these enzymes that have been credited with this role and are frequently over-expressed in tumors are the group of glutathione reductases, glutathione peroxidases, the cytosolic glutathione *S*-transferases (GSTs) and the metallothioneins [110–112] Common feature of the above enzymatic systems is the presence of cysteine units which render them potent free radical scavengers and function as effective detoxification system that play a pivotal role in the protection of cells against damage induced by free radicals [113,114]. It has been hypothesized that co administration of a substance blocking such redox systems could enhance the efficacy of hypericin PDT [115,116]. Furthermore, this cell response to oxidative stress is also related to the irradiation conditions. For example, regeneration of reduced glutathione (GSH) during dark intervals was considered to be responsible for the resistance of cells to PDT in an *in vitro* study with fractionated irradiation (light/dark intervals of 45/60 sec) [117] using A431 human epidermoid carcinoma cells.

In a recent *in vitro* study by Karioti *et al*. [118], hypericin and in a higher extent pseudohypericin inhibited in the dark thioredoxin reductase I and II. Interestingly, the polar pseudohypericin binded stronger than hypericin to the enzyme causing inhibition comparable to cisplatin. Dabrowski *et al*. [119], using human kidney 293 cells observed that increased glutathione *S*-transferases expression is inversely correlated with hypericin efficacy in PDT. Surprisingly, GSTP1-1 over-expression led to an increase in cellular uptake of hypericin, which suggested that this may result from direct sequestration of hypericn by GSTP1-1. It was also postulated that over-expression of GSTP1-1 caused a change in the expression of transport proteins, and thus altered the steady-state levels of intracellular hypericin. Alternatively, the oxidative stress caused by PDT may have led to higher levels of lipid peroxidation products and GSH conjugates. These, in turn, could compete with hypericin for transporter sites, thus leading to an increased intracellular concentration of hypericin. Finally, it was suggested that that cells overexpressing GSTP1-1 could protect neighboring cells from hypericin-dependent PDT by sequestering the photosensitizer.

In contrast, in another study depletion of intracellular glutathione reductase (>85%) in human prostate carcinoma cells *via* inhibition of γ-glutamyl-cysteine synthase had no effect on hypericin phototoxicity [120], thus precluding any direct oxidative involvement of H_2_O_2_. Also, there was no change in intracellular SOD activity immediately after hypericin irradiation.

In another *in vitro* study [121] in well-differentiated HK1 nasopharyngeal cancer cells a differential up-regulation of metallothionein in isoforms was observed: MT-1E and MT-2A isoform were up-regulated of six hours following PDT, with an approximately 50-fold rise in the expression level of MT-1E and a 15-fold increase of MT-2A. However, cells still succumbed to PDT-induced necrosis. It appears that the oxidative stress induced by PDT overwhelmed the antioxidant defense mechanism such as the alteration of MT levels in tumor cells.

### 6.3. Anti-angiogenic Antimetastatic Activity

As mentioned initially, PDT causes serious damages to the tumor vascular endothelial cells resulting to microvascular collapse. In molecular level, inhibition of the phosphorylation and activation of the ERK pathway has been reported [122]. Nevertheless, microvascular collapse may lead to a better tumor control, on one hand, but on the other hand, tumor hypoxia, upregulates several pro-angiogenic factors.

Bhuvaneswari *et al.* studied the angiogenic responses in a human bladder carcinoma xenograft model under short (0.5 h) and long (6 h) drug light intervals of hypericin-PDT treatment at 24 h and 30 days post treatment [123]. Short drug light interval PDT caused extensive vascular damage. In the contrary, long drug light interval hypericin-PDT induced the expression of angiogenic proteins, such as vascular endothelial growth factor (VEGF), tumor necrosis growth factor-α (TNF-α), interferon-α (IFN-α) and basic fibroblast growth factor (bFGF). Similarly, using another model (*in vivo* human nasopharyngeal xenograft model) the same group [124], made similar observations: VEGF levels were found to be higher when the tumors were treated at a 1 h drug-light interval compared to a 6 h interval, due to extensive vascular damage, whereas 72 h after hypericin-PDT, VEGF levels were upregulated indicating the initiation of regrowth in tumors. With the co-administration of the angiogenesis inhibitor, celebrex®, a downregulation of the human VEGF levels was observed, suggesting an alternative way to overcome the angiogenic post hypericin PDT effect and improve the outcome of hypericin-PDT in nasopharyngeal carcinomas. Neverthelless, the time of the initial administration of Cerebrex-post PDT is also an important factor for the tumor control [125].

The anti-angiogenic effects of hypericin seem to be irrelevant to the presence or not of light. In an *in vitro* study by Martinez-Poveda *et al*. [126], the effects of non-photoactivated hypericin in key steps of angiogenesis, (including proliferation, tubular formation, extracellular matrix protease production, migration and invasion) were studied on bovine vascular endothelial cells. Hypericin in the dark inhibited moderately endothelial cell proliferation with an IC_50_ of 10 μM (*vs*. 13 nM of photoactivated hypericin). Most notably, it inhibited the formation of tubular-like structures on Matrigel in the same range of concentration as the photoactivated hypericin and in lower or in the same range of concentrations of other known inhibitors. Hypericin also presented inhibitory effects on migration and invasion of endothelial cells by causing a remarkale decrease in the levels of extracellular matrix degrading urokinase but not on those of matrix metalloproteinases MMP-2. *In vivo* studies also confirm the antimetastatic activity of hypericin in the dark [127]. Treatment with hypericin significantly reduced growth rate of metastases in two murine models: breast adenocarcinoma (DA3) and squamous cell carcinoma (SQ2). Hypericin was reported to accumulate in primary and metastatic tumors and its content in lungs bearing metastases was measured to be approximately 2-fold higher than in the lungs of healthy animals. Long-term animal survival in DA3 tumor-excised groups increased from 15.6% in controls to 34.5% following supplementary treatment with hypericin, whereas in mice bearing SQ2 tumor metastases, therapy with hypericin increased animal survival from 17.7% in controls to 46.1%.

## 7. Antidepressive Effects of Naphthodianthrones

Currently, from all the scientific literature it is generally accepted that St. John’s wort preparations have a good profile of efficacy in mild to moderate depression and high tolerability. For this reason literature data concerning the antidepressive activity of isolated naphthodianthrones are limited.

Early biochemical studies reported that hypericin is an inhibitor of MAO-A and MAO-B activity [128]. The concentrations that produce 50% inhibition (IC_50_) ascertained by Suzuki [128] are 100- to 1,000-fold higher than accessible Cmax values of hypericins after oral administration of SJW extracts.

Hypericin can bind to the GABA_A_ receptors and at the 5-HT_1_ receptor [129], while has no significant affinity (<10% inhibition) for the following receptors: adenosine (A_1_ or A_2_), adrenergic (α_1_, α_2_ or β_1_), angiotensin (AT_1_), central BZD, cloned human bradykinin (B_2_), D_1_, GABA, cloned human tachykinin NK_1_, NPY, opioid, PCP, glucocorticoid, or vasopressin (V_1_). It has weak affinity (10–40% inhibition) for CCK_A_, D_2_, cloned human endothelin ET _A_, NMDA, central H_1_ and central nAChR sites. The two highest levels of inhibition (>40%) are at (non selective) muscarinic cholinergic receptors (49%) and at (non selective) σ receptors (about 48%) [130]. The affinity for NMDA receptor has also been confirmed by another investigation [131]. Hypericin and pseudohypericin inhibit the binding to rat D_3_ and D_4_ receptors, hypericin, but not pseudohypericin, inhibits binding to rat β1- and rat β2-adrenoceptors. Neither substance affects the SERT, the 5-HT receptor or the NA transporter [132]. Surprisingly, a 5-HT concentration increase was observed in the hypothalamus of rats after administration of pure hypericin for eight weeks, but not for two weeks administration. In contrast, the concentration of NA was reduced in the hippocampus after two weeks of daily administration of hypericin, whereas in the hypothalamus hypericin had no effect on NA levels. Nevertheless, hypericin showed efficacy in the Porsolt test, a sensitive depression animal model [32].

Hypericin inhibits the enzyme dopamine-β-hydroxylase with an IC_50_ of 3.8 μmol/L [133,130], whereas tyrosinase and tyrosine decarboxylase are not influenced by 1 to 10 μM hypericin [134]. Pseudohypericin also inhibits the enzyme dopamine-β-hydroxylase (IC_50_ = 3 μM) [130].

Hypericin increased 5-HT concentrations in the hypothalamus after chronic treatment for 8 weeks and exhibited neuroendocrine effects in rats by decreasing plasma levels of adrenocorticotropic hormone and corticosterone after 14 days of oral treatment [132,135]. It also affected the transcription of genes involved in the regulation of the hypothalamic–pituitary–adrenal axis: mRNA levels of both corticotropin-releasing factor (CRF) and serotonin 5-HT1A receptor were decreased in the hypothalamic paraventricular nucleus and in the hippocampus, respectively [136]. Since corticotropin-releasing factor (CRF) seems to be a major determinant in the regulation of the hypothalamic–pituitary–adrenal activity *via* activation of CRF_1_ receptors, hypericin and pseudohypericin were assayed for their antagonist activity on the CRF_1_ receptor. Pseudohypericin selectively antagonised CRF (KB 0.76 AM) and the authors concluded that pseudohypericin is the only real CRF_1_ receptor antagonist [137].

## 8. Antiviral Activity

Hypericin and in lesser extent pseudohypericin have been the subject of intense research for their antiviral properties. A detailed review article by Kubin *et al.* [77] has covered this topic. In general, hypericin has exerted antiviral activity *in vitro* against a great variety of viruses. It has shown virucidal activity by inhibiting viral infectivity in a hypericin-preincubation and light-dependent inactivation reaction and/or (II) antiviral activity by inhibiting viral replication in cell cultures. This activity is influenced a great deal by the presence of light and oxygen. Both factors seem to be important for the antiviral/virucidal properties of the molecule.

Further studies in recent years have shown that hypericin inhibited human cytomegalovirus *in vitro* [138], inhibited the adsorptive ability of Foot-and-Mouth virus (FMDV) to host cells *in vitro* on a model of BHK-21 cells with maximal inhibitory rate of 59.72% [139] and had a dose-dependent activity against porcine reproductive and respiratory syndrome virus (PRRSV) in amodel of Marc-145 cells [140]. The maximum of activity was observed (cell survival rate and inhibitory rate) when the Marc-145 cells were pretreated with hypericin and then challenged with the viruses. The authors do not comment on the light conditions. In another study [141] on HHV-6-infected lymphoblasts, hypericin was found completely inactive; again the light conditions are not described. Some interaction studies of hypericin with the with the human immunodeficiency virus-1 reverse transcriptase [142] and HIV-1 protease [143] have been reported. However, as already highlighted by the review of Kubin [77], administration of hypericin on HIV-1 and hepatitis C virus patients gave dissapointing results, since no antiviral effect was observed, and instead, in the dosage used, patients showed phototoxicity. This contradiction between *in vitro* an *in vivo* studies could be explained in terms of light irradiation. The absence of light in many regions of the body limits the use of hypericin as a therapeutic compound for the treatment of viral infections *in vivo*.

## 9. Antimicrobial Activity

The traditional use of St John’s Wort includes the treatment for bacterial infections, respiratory conditions, skin wounds, peptic ulcers and inflammation [144]. Several antimicrobial assays have been carried out with extracts of different *Hypericum* sp. [145,146]. Unfortunately, data concerning the activity of isolated components are scarce and even more, there are no data concerning the influence of light or oxygen on the activity of hyperin or pseudohypericin. During an antibacterial evaluation of *H. triquetrifolium* Turra [147], bioguided fractionation led to the identification of quercetin and 13,118-biapigenin as the substances responsible for the week antibacterial effect. Recently, on the other hand, hypericin and emodin isolated from an endophytic fungus of *H. perforatum* were assayed on a panel of laboratory standard pathogenic control strains, including fungi and bacteria. Both hypericin and emodin possessed antimicrobial activity comparable to authentic standards [17]. Furthermore, as already mentioned above, synthetic cationic derivatives of hypericin showed after irradiation strong antibacterial activity against the strain *Propionibacterium acnes.*

## 10. Other Activities

### 10.1. Ophthalmologic Applications

The antiangiogenic effects of hypericin have been investigated for their possible exploitation in ophthalmologic applications and in particular retinopathy. In an *in vitro* study [148] of a mouse model of oxygen-induced retinopathy, administration of hypericin inhibited retinal neovascularization, but did not affect the area of retinal vasoobliteration. Most importantly, experiments were carried out in room conditions, and therefore without photoactivation of hypericin. Further *in vivo* studies [122] using rat eyes in several ocular models, demonstrated a strong angiogenetic inhibitory activity of hypericin.

At molecular level, hypericin inhibited the activating phosphorylation of extracellular signal-regulated MAP kinases (ERK1/2) in human retinal pigment epithelial cells and in EA.hy926 cells, an endothelial hybridoma expressing endothelial cell properties. MT1-MMP activity in human microvascular endothelial cells was also inhibited. All *in vitro* and *in vivo* experiments were performed in the dark, without light activation. Concerning the *in vivo* tests, however, one has to consider that the retina is accessible to light and therefore it is possible that light may have contributed to the high anti-angiogenic activity potency of hypericin. This does not rule out the possibility that hypericin exerts antiangiogenic effects also in the dark, as discussed previously (see Section 6.2).

### 10.2. Anti-inflammatory

Light-activated pseudohypericin has been reported to inhibit the production of prostaglandin E_2_ (PGE_2_) [149], while hypericin has been reported to reduce of Croton-oil-induced ear oedema in mice in concentration (ID_50_ 0.25 μmol/cm^2^) comparable to that of indometacin (ID_50_ 0.26 μmol/cm^2^) [150].

### 10.3. Interaction with b-Amyloid Peptides

In an *in vitro* study by Sgarbossa *et al.* [151] hypericin at a concentration 10^−5^ M prevented or arrested the aggregation process of 1–40 b-amyloid peptides. Hypericin was reported to interact also with the early precursors of the b-sheet fibrils and/or protofibrils. More interestingly, hypericin was used to monitor (*in vitro*) the appearance of early aggregation states of b-amyloid peptides during the polymerization process.

On the contrary, in an *in vitro* study using mouse microglial cell lines, neither hypericin nor pseudohypericin were able to protect microglia against amyloid beta [Aβ (25–35) and Aβ(1–40)] induced cell death [152].

## Figures and Tables

**Table 1.** *In vitro* studies using Hypericin - PDT in different cancer cell lines.

**Cell culture**	**Light**		**Reference**
human umbilical endothelial cells and human glioma cancer cells U-87 MG & U-373 MG	+	sensitive only to photoactivated hypericin	[153]
human HepG2 cancer cells	+		[154]
hepatic hepatoblastoma HUH6, & HepT1 cells	+	severe alterations only after illumination	[155]
pediatric hepatocellular carcinoma HepG2 cells	+	severe alterations only after illumination	[155]
human lung SpcA1 cancer cells	+	light emitting diode as light source for photoactivation	[156]
human lung cancer cells A549	+		[157]
MDA231 human mammary carcinoma cells	+	light emitting diode as light source for PDT	[158]
human renal carcinoma cells	+		[159]
rhabdomyosarcoma cells and fibroblasts	+	nearly complete inhibition of cell proliferation only after photoactivation	[160]
